# Identification and Characterization of ShSPI, a Kazal-Type Elastase Inhibitor from the Venom of *Scolopendra Hainanum*

**DOI:** 10.3390/toxins11120708

**Published:** 2019-12-05

**Authors:** Ning Luan, Qiyu Zhao, Zilei Duan, Mengyao Ji, Meichen Xing, Tengyu Zhu, James Mwangi, Mingqiang Rong, Jiangxin Liu, Ren Lai

**Affiliations:** 1 Department of Zoology, College of Life Sciences, Nanjing Agricultural University, Nanjing 210095, China; 2017216016@njau.edu.cn (N.L.); 2017116050@njau.edu.cn (M.J.); 2017116048@njau.edu.cn (M.X.); 2017116049@njau.edu.cn (T.Z.); 2 Key Laboratory of Animal Models and Human Disease Mechanisms of Chinese Academy of Sciences/Key Laboratory of Bioactive Peptides of Yunnan Province, Kunming Institute of Zoology, Chinese Academy of Sciences, Kunming 650223, China; zhaoqiyu@mail.kiz.ac.cn (Q.Z.); duanzilei@mail.kiz.ac.cn (Z.D.); jams@mail.kiz.ac.cn (J.M.); 3 The National & Local Joint Engineering Laboratory of Animal Peptide Drug Development, College of Life Sciences, Hunan Normal University, Changsha 410081, China; 4 State Key Laboratory of Phytochemistry and Plant Resources in West China, Kunming Institute of Botany, Chinese Academy of Sciences, Kunming 650201, China; 5 Sino-African Joint Research Center, Kunming Institute of Zoology, Chinese Academy of Sciences, Kunming 650223, China

**Keywords:** centipede, kazal-type inhibitor, human neutrophils elastase, ShSPI

## Abstract

Elastase is a globular glycoprotein and belongs to the chymotrypsin family. It is involved in several inflammatory cascades on the basis of cleaving the important connective tissue protein elastin, and is strictly regulated to a balance by several endogenous inhibitors. When elastase and its inhibitors are out of balance, severe diseases will develop, especially those involved in the cardiopulmonary system. Much attention has been attracted in seeking innovative elastase inhibitors and various advancements have been taken on clinical trials of these inhibitors. Natural functional peptides from venomous animals have been shown to have anti-protease properties. Here, we identified a kazal-type serine protease inhibitor named ShSPI from the cDNA library of the venom glands of *Scolopendra hainanum*. ShSPI showed significant inhibitory effects on porcine pancreatic elastase and human neutrophils elastase with *Ki* values of 225.83 ± 20 nM and 12.61 ± 2 nM, respectively. Together, our results suggest that ShSPI may be an excellent candidate to develop a drug for cardiopulmonary diseases.

## 1. Introduction

Centipedes are predatory arthropods distributed on every continent except Antarctica. They prey mainly by secreting venom from the venom glands, which are connected to the first pair forcipules. Centipede venoms are comprised of proteins and peptides, which show a variety of biological activities [1]. More than 500 proteins and peptides in centipede venom have been found with diverse pharmacological properties, including the activity of platelet aggregation, anticoagulation, phospholipase A2 (PLA2), trypsin inhibition, ion channel inhibition, and antimicrobial [2,3,4,5,6,7]. Despite the clear clinical implications following envenomation by *Scolopendra hainanum* (http://arachnoboards.com/threads/scolopendra-hainanum.308202/), there is no related study on *S. hainanum* toxins, currently.

Elastase is a group of serine proteases that include the macrophage elastase, the fibroblast elastase, the neutrophil elastase, and the pancreatic elastase, which can not only cleave the important connective tissue protein elastin, but also facilitate the degradation of the extracellular matrix such as fibronectin; laminin; collagens III, IV, and VI; and proteoglycans. Human neutrophil elastase (HNE) is a serine protease (29 kDa) expressed by neutrophil upon activation, which can be secreted into the phagosome during phagocytosis or released during neutrophil necrosis [8,9]. In physiological condition, the activity of HNE is strictly regulated to a balance by several endogenous inhibitors, including elafin, serpins, α1-antitrypsin, and secretory leukocytes proteinase inhibitor. When out of control, HNE can cause severe diseases such as acute lung injury, acute respiratory distress syndrome, chronic obstructive pulmonary disease, and pulmonary fibrosis [9]. To stabilize these diseases and ameliorate symptoms, new and specific anti-proteases, especially elastase inhibitors, might be excellent candidates.

Numerous peptidic elastase inhibitors have been identified from the toxins of venomous animals [10,11], e.g., secapin from bee venom [12], BmKTT-2 from scorpion venom [13], AvCI from spider venom [14] and guamerin from leech secretions [15]. These elastase inhibitors exhibit potent inhibitory effects to elastase and provide a valuable source for new drug development. Although over 500 proteins or peptides with diverse pharmacological properties from the centipede venom have been discovered, there is no report about the elastase inhibitor from the centipede toxins.

In this study, we investigated a novel elastase inhibitor named ShSPI, which belongs to the atypical kazal-type proteases inhibitor and has the significant inhibitory effects on porcine pancreatic elastase (PPE) and HNE. Sivelestat is a specific HNE inhibitor, which has been reported to mitigate lung injury in several mouse models, including pulmonary fibrosis and acute lung injury [16,17]. Comparing to sivelestat, ShSPI demonstrates better inhibitory activity to elastases. Our results suggest that ShSPI may be an excellent candidate to develop the drug for elastase related diseases, such as cardiopulmonary diseases.

## 2. Results

### 2.1. Determination of the Primary Structure of ShSPI

**Figure 1:**
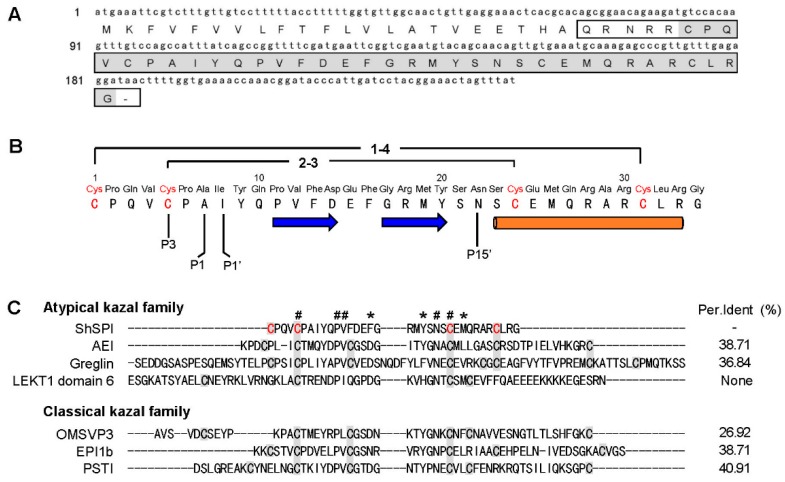
Primary structure of ShSPI. (**A**) cDNA encoding the precursor of ShSPI. The sequence without signal peptide is boxed. The mature form, named ShSPI, is indicated by grey color. (**B**) The primary structure of ShSPI. The disulfide bond pairing mode is C_1_–C_4_/C_2_–C_3_. ShSPI consists of a cystine-stabilized α-helical (CSH) motif formed by residues Ser-23 to Arg-33, and a two-stranded antiparallel β-sheet (strand 1, Pro-11 to Asp-14; and strand 2, Gly-17 to Tyr-20). The putative P1–P1′ sites were suggested using HNE as reference enzyme and the nomenclature of Schechter and Berger [18,19]. (**C**) Similarity of ShSPI to selected atypical kazal family and classical kazal family. The percent identity (Per.Ident) (%) of ShSPI with each sequence has been shown to demonstrate their sequence similarity. The cysteine residues in domains are shown in grey color. The conserved residues are marked with #, and residues with high similarity are indicated by asterisk.

A cDNA sequence encoding a precursor protein composed of 61 amino acid (aa) was found. A hypothetical signal peptide (22 aa), pro-peptide (-QRNRR-), and a mature peptide (34 aa) were identified (Figure 1A, marked by box) through online analysis (SignalP-5.0, http://www.cbs.dtu.dk/services/SignalP/). BLAST search indicated that the mature peptide named ShSPI (Figure 1A, marked by grey color) shares pair sequence similarity with other atypical kazal family (Figure 1C). The amino acid sequence of ShSPI is indicated in Figure 1B: CPQVCPAIYQPVFDEFGRMYSNSCEMQRARCLRG.

### 2.2. Refolding of ShSPI

**Figure 2:**
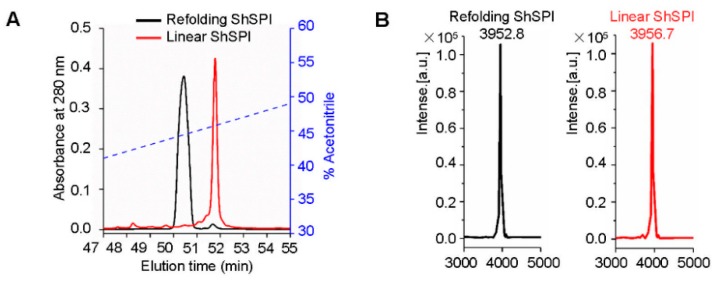
Refolding of ShSPI. (**A**) Linear ShSPI was synthesized and refolding analysis was performed by C_18_ HPLC. (**B**) MALDI-TOF MS was used to confirm the purity of ShSPI to be higher than 95%. Average molecular weight of refolding ShSPI and linear ShSPI are 3952.8 and 3956.7 Da, respectively, which indicated that two disulfide bonds were formed successfully.

We chemically synthesized linear ShSPI and refolded its two disulfide bridges with the glutathione redox system (Figure 2A). C_18_ reverse phase-high performance liquid chromatography (RP-HPLC) was conducted to purify the refolded ShSPI, with the elution of indicated gradients of acetonitrile (containing 0.1% (v/v) trifluoroacetic acid) at a flow rate of 1.5 mL/min. Matrix-assisted laser desorption/ionization time-of-flight mass spectrometry (MALDI-TOF MS) was applied to determine the purity of peptides to be higher than 95% (Figure 2B). Consistent with the predicted molecular weight (MW) of ShSPI, the observed MW of refolded ShSPI was 3952.7 Da, indicating that the two disulfide bridges have been refolded successfully.

### 2.3. ShSPI Inhibits Elastase Potently and Selectively

**Figure 3:**
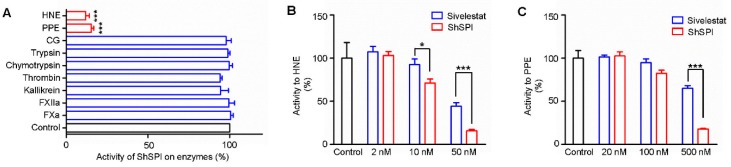
ShSPI inhibits elastase selectively and potently. (**A**) ShSPI (500 nM) was tested against nine different serine proteases, and triplicates tests were performed. The activity of ShSPI on enzymes are illustrated with percentage. Compared with sivelestat, a potent elastase inhibitor, ShSPI could affect more activity of HNE (**B**) and PPE (**C**) at the same concentration. Each error bar shows mean ± SEM. *p* values were determined by student’s t test (*p < 0.05, ***p < 0.001).

Based on the sequence alignment analysis, we speculate that ShSPI may contain inhibitory activity on some proteases. The enzyme activity of ShSPI to a diversity of proteases was assayed. ShSPI (500 nM) exhibited significant inhibitory activity on porcine pancreatic elastase (PPE) and human neutrophil elastase (HNE), while it showed no inhibitory activity against factor Xa (FXa), factor XIIa (FXIIa), kallikrein, thrombin, trypsin, chymotrypsin, or cathepsin G (CG) (Figure 3A).

Sivelestat (also known as ONO-5046), which is a potent elastase inhibitor, has been used as a clinical drug [20]. It has been reported that sivelestat has little inhibitory activity to PPE and strong inhibitory activity to HNE with a *Ki* value of 200 ± 20 nM [21]. We compared the inhibitory activity to PPE and HNE of ShSPI and sivelestat. As illustrated in Figure 3B,C, both ShSPI and sivelestat significantly inhibited the activity of PPE and HNE in a dose-dependent manner. However, ShSPI showed better inhibitory effect on PPE and HNE than sivelestat. At the concentration of 500 nM, the inhibitory rate of ShSPI and sivelestat on PPE was about 18% and 65%, respectively. At the concentration of 50 nM, ShSPI and sivelestat exhibited an effect on HNE with an inhibitory rate of 16% and 44%, respectively.

### 2.4. Enzymatic Kinetics of ShSPI on Elastase

**Figure 4:**
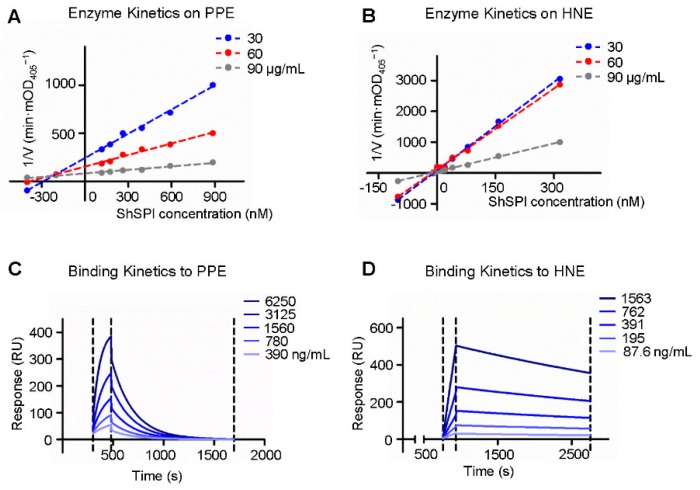
The enzyme and binding kinetics of ShSPI to elastase: the Dixon plots of ShSPI to PPE and HNE are indicated, respectively (**A**,**B**); and surface plasmon resonances of ShSPI to PPE and HNE were performed, respectively (**C**,**D**). ShSPI was immobilized on a sensor-CM5 chip and PPE or HNE was injected for 180 s at different concentrations, dissociation of the ShSPI-elastase complex was monitored more than 1000 s. “Langmuir binding” fitting model was used to fit the data.

The enzymatic kinetics of ShSPI on elastase was further explored; varying doses of ShSPI (from 0 to 885 nM) and elastase colorimetric substrate (30, 60, and 90 μg/mL) were used. The Dixon plot analysis showed that linear curves converged on the x-axis, suggesting that ShSPI is a non-competitive inhibitor of elastase (Figure 4A,B) and the *Ki* values to PPE and HNE were 225.8 ± 20 nM and 12.6 ± 2 nM, respectively (Table 1).

### 2.5. Binding Kinetics of ShSPI on Elastase

To verify the direct interaction of ShSPI and elastase, surface plasmon resonance (SPR) biosensor technology was used to determine the binding affinity of ShSPI to HNE and PPE. The ability of ShSPI binding to elastase was reflected by the response values (RU) that were directly recorded by the Biacore 3000 instrument (GE Healthcare Life Sciences, Pittsburgh, PA, USA). As shown in Figure 4C,D, the RU increased with increasing elastase (HNE or PPE) concentration, which indicated that ShSPI binds to elastase in a concentration dependent manner.

The equilibrium dissociation constant (K_D_, M) of ShSPI to PPE was 1.0 × 10^−7^ and to HNE was 4.2 × 10^−8^ (as listed in Table 1). The binding of ShSPI with HNE was stronger than that with PPE, which is consistent with the protease inhibition assays.

### 2.6. The NMR Structure of ShSPI

**Figure 5:**
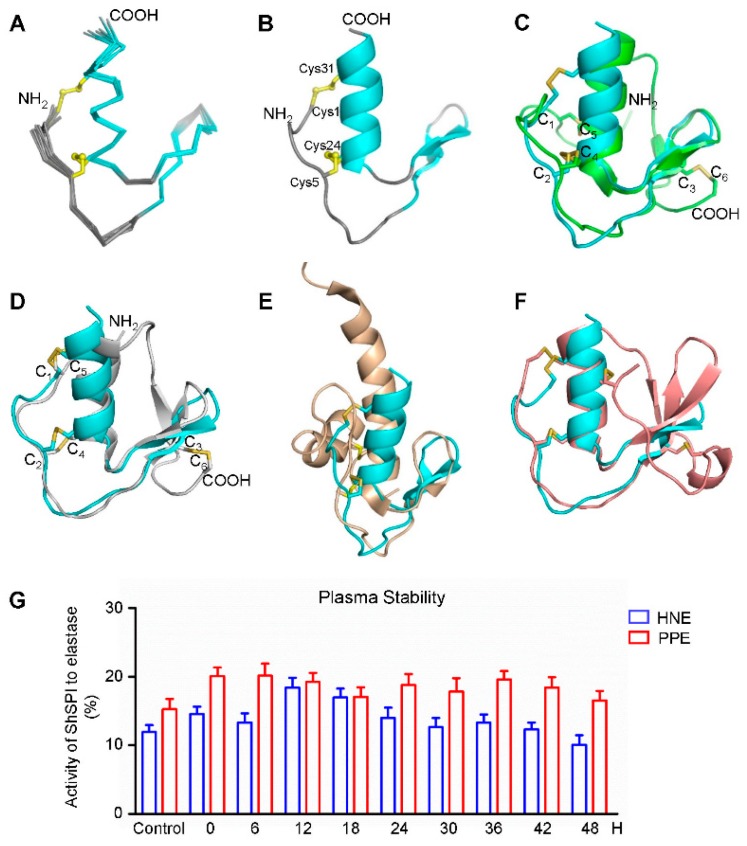
The nuclear magnetic resonance (NMR) structure and plasma stability of ShSPI. The disulfide bonds’ connectivity is shown in yellow ball-and-stick format and color-coded (**A**–**F**). (**A**) Backbone traces of the ShSPI structural ensemble, with the secondary structure region (shown in cyan) and the disorder loops (indicated in gray). (**B**) Cartoon diagram of ShSPI illustrating the locations of α-helix and anti-parallel β-sheets. The cysteine residues are labeled as ball-and stick format. (**C**) Superposition of the structure of ShSPI (cyan) and silver pheasant ovomucoid third domain (OMSVP3, classical kazal-type) (green). (**D**–**F**) Comparison of ShSPI (cyan) with atypical Kazal inhibitors, including: anemonia elastase inhibitor (AEI) (grey) (**D**); domain 6 of the LEKT1 (wheat tints) (**E**); and greglin inhibitor (**F**). Visible N- and C-termini in these orientations are labeled NH_2_ and COOH, respectively. Domain 6 of the LEKT1 (**E**) is rotated approximately 90° along the vertical axis compared with Figure 5D for the clarity of the first disulfide bond. (**G**) Plasma stability was assayed by the enzyme inhibitory activity of ShSPI to PPE and HNE, which is consistent with the activity of ShSPI without incubated with human plasma. Data represent three independent experiments.

The three-dimensional structure of the ShSPI peptide was determined using two-dimensional (2D) nuclear magnetic resonance (NMR) spectroscopy. The chemical shift analysis was used to identify the disulfide-bonded or free-state cystein, and cis- or trans-conformation of the proline. The chemical shifts of the cysteins Cβ are all between 35.85 and 40.98 ppm, indicating that the disulfide bonds were formed. The initial structure calculations only with NOE restraints demonstrated the Cys-1 and Cys-31, and Cys-5 and Cys-24 disulfide bond format. The Cγ values of prolines are 27.89 ppm (Pro2), 27.38 ppm (Pro6), and 27.36 ppm (Pro11), respectively, indicating all of the prolines are in trans-conformation [22], while the Cγ values for prolines with cis-conformation fall at 24.4 ± 0.7 ppm. The unambiguous and strong H(i)-Hδ(i+1: Pro)(dαδ) nuclear overhauser effect (NOE) signals in the NOESY spectra also confirmed the trans-conformation. The H(i)-Hδ(i+1: Pro)(dαδ) or Hα(i)-Hα(i+1: Pro)(dαα) NOEs of Pro (i+1) and its immediate preceding residue (i) were used to assign the configuration of Pro residues. The strong dαδ NOEs indicates the trans-configuration while strong dαα NOEs indicates the cis-configuration. The structural statistics for the final 15 structure ensembles (Figure 5A,B) are given in Table 2.

The structure of ShSPI consists of a cystine-stabilized α-helical (CSH) motif formed by residues Ser-23 to Arg-33, and a two-stranded antiparallel β-sheet (strand 1, Pro-11 to Asp-14; and strand 2, Gly-17 to Tyr-20). The N-terminal loop is stabilized by the formation of disulfide bonds. The NOE pattern demonstrates that Cys-1 (C_1_) forms a disulfide bond with Cys-31 (C_4_), while Cys-5 (C_2_) with Cys-24 (C_3_). This is further evidenced by the partial reduction and MS data (data not shown). Taken together, ShSPI contains the C_1_–C_4_ and C_2_–C_3_ disulfide bond connectivity (Figure 1B).

### 2.7. ShSPI is an Atypical Kazal Domain and the Minimal Functional Unit

As the serine protease inhibitors, the canonical kazal domain is composed of one α-helix and three-stranded anti-parallel β-sheet, six cysteins forming three disulfide bonds with the pattern of C_1_–C_5_, C_2_–C_4_, and C_3_–C_6_. Kazal domains with two disulfide bonds and the absence of the third and six cysteines were referred as atypical kazal domains [23]. The ShSPI belongs to this class. The structure superimposition with the canonical kazal domain OMSVP3 (pdb: 1IY5) is demonstrated in Figure 5C, which shows that the tertiary structure of ShSPI resembles the core structure of canonical kazal domain.

To gain insights into the difference between ShSPI and other atypical kazal inhibitors, we superimposed the three-dimensional structures of ShSPI with other reported atypical kazal domain structures, including anemonia elastase inhibitor (AEI, pdb: 1Y1B) (Figure 5D), domain 6 of LEKT1 (pdb: 1H0Z) (Figure 5E), and Greglin (pdb: 4GI3) (Figure 5F). Compared with ShSPI, other atypical kazal domains show either NH_2_- or COOH-terminal long or short extensions. The structural comparison demonstrates that the ShSPI motif with 34 residues is the core structure contained by all of the atypical kazal domains. Meanwhile, ShSPI contains very short sequence with only three residues between the first two cysteines, instead of 6–13 residues in the typical kazal domains (Figure 1C, bottom). Together with its activity, to the best of our knowledge, the ShSPI could be regarded as the minimal functional unit.

### 2.8. Point Mutations Confirm the Important Residues of ShSPI

The sequence analysis of the kazal-family shows that P3, the second conserved cysteine residue, and P15′ with the residue asparagine are highly conserved, while the remaining ten contact residues are hypervariable. The numbering is according to the nomenclature of Schechter and Berger and using HNE as reference enzyme [18,19]. We supposed that ShSPI conforms to this regular pattern. The ShSPI mutant C5A (P3) and N22A (P15′) of ShSPI are not soluble or show no activity (*Ki* > 1 mM) (Table 3), indicating that the point mutation disrupts the global fold and the mutant could not fold properly (data not shown). These results confirm the essential importance of residues in the P3 and P15′ positions.

To further confirm the important residues for the interaction between ShSPI and HNE, point mutation method was applied. Through the structural analysis, several ShSPI mutants were synthesized, including P2A, Q3A, V4A, P6A Y9A, Q10A, S21A, N22A, and E25A. The inhibitory effects of these mutants to HNE were tested. Mutants Y9A, N22A, and E25A did not show any inhibitory activity to HNE, demonstrating that these three residues are vital for the inhibitory activity of ShSPI. Other residues of ShSPI, such as Pro2, Gln3, and Pro6, are also important for the inhibitory to elastase. The single mutation significantly decreased their HNE activity. The *Ki* values of these mutants are listed in Table 3.

### 2.9. ShSPI Showed Plasma Stability

The structural information shows that ShSPI is the minimal functional unit and the folding is stabilized through the two intra-disulfide bonds and the tertiary structure. To investigate the stability of ShSPI in physiological condition, ShSPI was co-incubated with human plasma over 48 h. ShSPI was found to be remarkably stable in human plasma, with no significant degradation over 48 h. Compared with the basal inhibitory activity, the inhibitory percentage to HNE and PPE was still around 12% and 17%, respectively (Figure 5G). Stability of ShSPI in plasma could support the hypothesis that ShSPI could be stable and affect strongly in vivo.

## 3. Discussion

Venomous creatures are considered to be a very distinctive class of species among animals [22]. Venomous animals are equipped with venom glands and venoms, which provide them outstanding advantages for their existence [24,25,26]. Toxins from centipedes have been well studied. Some of them have been established as important tools for predation, and some of them are useful candidates for drug design [22,27]. To the best of our knowledge, as a novel member of centipedes distributed only in Hainan and Guangxi province, China, the bioactive peptides from venom of *S. hainanum* have not been reported [26,28]. For the first time, we identified a new serine protease inhibitor (SPI) from *S. hainanum*, named as ShSPI and characterized as an atypical kazal motif. Through the sequence and structural analysis, the ShSPI is considered as the minimal functional unit. The core motif includes the reactive loop with extended conformation and appropriate backbone geometry, the two disulfide bonds-stabilized α-helical (CSH) motif, and two-stranded anti-parallel β-sheet. It is missing two cysteine residues and the corresponding disulfide bond is not essential for inhibitor reactivity and stability [23].

SPIs are widely distributed in a variety of species [29,30]. They are able to inhibit the catalytic activity of proteolytic enzymes and guarantee the inhibiting proteolysis of proteinase, which are important in poisonous animals’ biological processes for survival [31,32]. Among kazal-type SPIs, most domains described so far contain six highly conserved cysteine residues, with the formation of three intra-domain disulfide bonds. In our study, ShSPI has only two disulfide bonds, thus belongs to atypical kazal family. However, the tertiary structure of ShSPI resembles the core structure of canonical kazal domain, which may provide the primary interaction site of ShSPI to proteinases. Classical SPIs with multiple kazal domains show broad-spectrum inhibition on serine proteinases such as trypsin, subtilisin, thrombin, chymotrypsin, and elastase potently. Despite the single kazal domain instead of multiple kazal domains in other SPIs, ShSPI has a stable structure which exhibits strong activity against elastase. In consistence with this, our results show that ShSPI specifically inhibits elastase with *Ki* values of 225.83 ± 20 nM to PPE and 12.61 ± 2 nM to HNE.

Other atypical kazal family inhibitors have shown narrower specificity compared to the classical kazal family. However, ShSPI is more specific. The structural similarity and difference within these inhibitors may give some explanation. All of the atypical kazal family inhibitors have the similar core structure, while ShSPI does not have the additional NH_2_- or COOH-terminal extensions and contains very short sequence with only three residues between the first two cysteines, instead of 6–13 residues in the typical kazal domains. It was reported that, when rigidity increases near the reactive site, the inhibitor is more likely to show narrower specificity. For example, compared with wild-type AEI, the AEI analog (just one aa between the first two cysteines) did not show inhibitory activity against PPE, while it retained almost the same potent inhibitory activity toward *Streptomyces griseus* protease B [33]. As a result, the characteristics of ShSPI can make it easier to reach the interaction site of elastase, and ShSPI could be a potential probe to study the interaction characteristics between SPIs and enzymes, which could provide us a clue to design the inhibitors with high specificity and selectivity.

Sivelestat, the selective neutrophil elastase inhibitor has been safely used in specific clinical scenarios, including refractory Kawasaki disease [34] and acute lung injury [16,35]. It also has been found that sivelestat can improve the survival in the clinical relevant animal models of sepsis, in which not only did damage occur in the lung, but also other organs including kidneys, liver, and heart were impaired [36,37,38,39]. From the results, we found that ShSPI exhibited better effects on the inhibitory of PPE and HNE than that of sivelestat at the same concentration. ShSPI might be an excellent candidate to develop the drug for the diseases sivelestat treated.

In conclusion, a potent elastase inhibitor, named ShSPI, was identified and characterized from the centipede *S. hainanum* for the first time. ShSPI has a stable three-dimensional structure and strongly binds to the catalytic site of elastase, which confirmed the inhibitory effect on elastase. Our findings have important implications for the design and development of new drugs for diseases related with the destruction of HNE.

## 4. Materials and Methods

### 4.1. RNA Extraction and cDNA Synthesis

The venom glands of *S. hainanum* were collected. Total RNA was isolated by using TRIzol reagent (15596018, Invitrogen, Waltham, MA, USA), and cDNA was reverse-transcribed using SuperScript II Reverse Transcriptase and oligo(dT)12−18 primer with the SMART™ PCR cDNA synthesis kit (Clontech, Palo Alto, CA, USA). The clones of cDNA (>350 bp) were selected and sequenced by Sangon Biotech (Shanghai, China).

### 4.2. Peptides Synthesis and Refolding

Linear ShSPI and its mutant peptides were synthesized by GL biochem (Shanghai, China). Single site mutation peptides were designed according to the method described [40].

Peptides were refolded in a solution (pH 7.2) containing 10 mM glutathione (V900456, Sigma, Saint Loui, MO, USA) and 100 mM oxidized glutathione (V900363, Sigma, Saint Loui, MO, USA). The refolding solution was placed at 28 °C for 24 h. Then, we separated the different fractions using Reverse Phase-High Performance Liquid Chromatography (RP-HPLC) techniques and C_18_ column (Waters, Milford, MA, USA, 5 μm particle size, 250 mm × 4.6 mm). Matrix-assisted laser desorption/ionization time-of-flight mass spectrometry (MALDI-TOF MS, Autoflex speed TOF/TOF, Bruker Daltonik GmbH, Bruker Corporation, Germany) was performed to determine the purity of the peptides, and refolded ShSPI with purity higher than 95% was collected for further research.

### 4.3. Assignment of the Disulfide Bonds

Refolded ShSPI (1 mg) was dissolved in 100 μL of 6 M guanidine hydrochloride (pH 6.0, G4505, Sigma, Saint Loui, MO, USA), and incubated for 30 min at 37 °C. Afterwards, 100 μL of 0.1 M TCEP (75259, Sigma, Saint Loui, MO, USA) were added to the sample and maintained for 3 min at 37 °C, and then 800 μL of 0.1% TFA-H_2_O were added immediately. Fractions were purified by using C_18_ RP-HPLC (Waters Corporation, 34 Maple Street Milford, MA, USA) with a linear acetonitrile gradient (0–60% over 60 min). The intermediate peptides were gathered, lyophilized, and alkylated with 0.5 M iodoacetamide (pH 8.3, I1149, Sigma, Saint Loui, MO, USA) for 1 min at 25 °C. Alkylated peptides were isolated and desalted by C_18_ RP-HPLC, and Edman degradation was performed on a Shimadzu protein sequencer (PPSQ-31A, Shimadzu, Nakagyo-ku, Kyoto 604-8511, Japan).

### 4.4. Protease Assays

Human neutrophils elastase (HNE, E8140), porcine pancreatic elastase (PPE, E0127), cathepsin G (CG, C4428), human trypsin (T6424), α-chymotrypsin from human pancreas (C8946), and thrombin from human plasma (T4393) were all purchased from Sigma-Aldrich (USA). Factor Xa (FXa, HFXa 1011) and Factor XIIa (FXIIa, HFXIIa 1212a) were from Enzyme Research Laboratories (South Bend, IN, USA). Kallikrein from human plasma (420307) was from Merck Millipore (Billerica, MA, USA).

Substrate for HNE and PPE (MeOSuc-Ala-Ala-Pro-Val-pNA, 324696) was purchased from Merck Millipore (USA). Substrates for CG (Succinyl-Ala-Ala-Pro-Phe-pNA, 573462), trypsin (Nalpha-Benzoyl-L-arginine 4-nitroanilide hydrochloride, B3133), FXa (CH3OCO-D-CHA-Gly-Arg-pNA-AcOH, F3301), chymotrypsin (N-Succinyl-Gly-Gly-Phe-pNA, S1899), thrombin (β-Ala-Gly-Arg-pNA diacetate, T3068), and kallikrein (Z-Phe-Arg 7-amido-4-methylcoumarin hydrochloride, C9521) were purchased from Sigma-Aldrich (USA).

As mentioned in our previous study [29], experiments for protease inhibitory activity were assayed in buffer (0.05 M Tris-HCl, pH 7.8) at 37 °C. The proteases (FXa, 0.1 nM; FXIIa, 10 nM; kallikrein, 400 nM; thrombin, 10 nM; chymotrypsin, 400 nM; trypsin, 10 nM; CG, 0.02 U/mL; HNE, 0.001 U/mL; and PPE, 1 μg/mL) and varying doses of ShSPI or sivelestat (MB1318, Meilun Biotehmology, Dalian, China) were pre-incubated for 10 min at 37 °C. At the end of the incubation, the reaction was initiated by the addition of the exclusive substrate solution and monitored by recording the absorbance at 405 nm every 30 s for 30 min. Enzyme activity in each well was calculated based on the initial slope of the reaction curve, and percentage of the initial slope of the uninhibited reaction was expressed. The inhibition constant *Ki* value was determined according to the Dixon method [41].

### 4.5. Surface Plasmon Resonance (SPR) Biosensor Analysis

Sensor chips CM5 (BR100012), 10 mM acetate buffer (BR100350, pH 4.0), amine coupling kit (BR100050), and HBS-P buffer (BR100826, used as running buffer) were all obtained from GE Healthcare Bio-sciences AB (Uppsala, Sweden). Data were collected and analyzed by BIAcore System (Version 3000, GE Healthcare Life Sciences, Pittsburgh, PA, USA) and BIA evaluation software (Version 3.0, GE Healthcare Life Sciences, Pittsburgh, PA, USA).

Before testing, ShSPI (100 μg/mL in acetate buffer, pH 4.0) was immobilized to the surface of CM5 chip. Following a standard amine coupling chemistry, running buffer was used to reach equilibration. HNE or PPE (doubling dilution) was injected over the immobilized ShSPI at a flow rate of 10 μL/min, and the association rate (k_a_, M^−1^s^−1^) was determined. After 3 min, running buffer was applied to the surface, and the dissociation rate (k_d_, s^−1^) was determined over 15 min for PPE and 30 min for HNE, and another 500 μL of running buffer were injected at a flow rate of 20 μL/min to rebalance the chip. The equilibrium association constant (K_A_, M^−1^) and the equilibrium dissociation constant (K_D_, M) were determined by k_a_/k_d_ and k_d_/k_a_, respectively [42].

### 4.6. NMR Experiments

The ShSPI nuclear magnetic resonance (NMR) samples contained ~1.5 mM protein in a buffer containing 25 mM HEPES, pH 6.8, 50 mM KCl, and 5 mM DTT in either 90% H_2_O/10% D_2_O or in 100% D_2_O. NMR data were collected at 298 K on a Bruker Avance 800 MHz spectrometer. Two-dimensional (2D) homonuclear total correlated spectroscopy (TOCSY) was recorded with mixing times of 60 and 80 ms. The data for t1 × t2 were 512 × 2048. 2D nuclear overhauser effect spectroscopy (NOESY) was collected for sequential residue assignments, with mixing time of 100, 200, and 300 ms. The data for t1 × t2 were 1024 × 4096. In addition, 2D ^1^H-^13^C heteronuclear single-quantum correlation spectroscopy (HSQC) was carried out with 2048 × 4096 complex points at natural abundance. ^1^H-^13^C TOCSY-HSQC spectra were recorded to help the sequential assignment [43], with the recording data of 1042 and 4096 complex points, respectively.

To demonstrate the hydrogen bonds within the peptide, hydrogen–deuterium (H/D) exchange experiments were also collected. D_2_O was used to dissolve the lyophilized peptide. A set of 1D spectra was collected. The time points included 20 min, 30 min, 1 h, 2 h, 4 h, 8 h, 24 h, and 48 h, respectively. When the H/D exchanged for 8 h, the TOCSY spectrum was also recorded. We used NMRPipe [44] and SPARKY (https://www.cgl.ucsf.edu/home/sparky) to process and analyzed the data.

The NOE were analyzed and assigned manually, and then converted into distance constraints. The program xplor-NIH [45] was used to calculate the structure. The quality of the NMR ensembles was evaluated by MolProbity [46].

### 4.7. Plasma Stability

Lyophilized human plasma (400100, Cayman, Ann Arbor, MI, USA) was resuspended in sterile water according to the instruction, then ShSPI was added to each equivalent volume with a final concentration of 500 nM, and samples were co-incubated at 37 °C for two days. Triplicate samples were collected at selected time points (0, 6, 12, 18, 24, 30, 36, 42, and 48 h) and protease activity was tested as described above.

### 4.8. Statistical Analysis

Experimental results were tested statistically by an unpaired two-tailed student’s t-test and expressed as mean ± SEM by GraphPad Prism (Version 5.0, GraphPad Software, San Diego, CA, USA). Data were considered statistically significant when *p* < 0.05.

## Figures and Tables

**Table 1:** Binding kinetics and affinity of ShSPI to elastase.

Compound		ShSPI	
Enzyme Injected		PPE	HNE
Enzyme kinetics	*Ki* (nM)	225.83 ± 20	12.61 ± 2
Binding Kinetics	k_a_ (M^−1^s^−1^)	4.5 × 10^4^	4.5 × 10^3^
	k_d_ (s^−1^)	4.7 × 10^−3^	1.9 × 10^−4^
	K_A_ (M^−1^)	9.7 × 10^6^	2.4 × 10^7^
	K_D_ (M)	1.0 × 10^−7^	4.2 × 10^−8^

**Table 2:** Structural Statistics of the ShSPI domain (15 structures).

Experimental Restraints of ShSPI (C1–G34)	
NOE distance restraints	448
Intra-residue	78
Sequential	167
Medium range (2 ≤ |i–j| ≤ 4)	100
Long range (|i–j| ≥ 5)	103
Hydrogen bonds constraints	4
**Energies (kcal/mol)**	
Bonds	4.99 ± 0.39
Angles	39.88 ± 1.53
Improper	5.34 ± 0.35
Van del Waals (repel)	17.13 ± 2.00
NOE	30.16 ± 4.08
**r.m.s** **. deviations from idealized ^1^**	
Bonds (Å)	0.003 ± 0.000
Angles (deg)	0.521 ± 0.010
Improper (deg)	0.347 ± 0.012
NOE	0.048 ± 0.003
**Ramachandran Plot**	
Most favored regions	93.8%
Additional allowed regions	6.2%
**Mean pairwise RMSD (ShSPI P2-R33) (Å)**	
Backbone	0.49 ± 0.10
Heavy Atoms	1.16 ± 0.20

^1^ Quality of the structures was evaluated by MolProbity.

**Table 3:** Enzyme inhibitory activity of mutants of ShSPI to HNE.

*Ki* Value of ShSPI to HNE: 12.61 nM					
Position ^2^	Mutants	*Ki* (nM)	Position ^2^	Mutants	*Ki* (nM)
P6	P2A	100.56	P3	C5A	insoluble
P5	Q3A	113.74	P2′	Y9A	>1 mM
P4	V4A	13.16	P15′	N22A	insoluble
P2	P6A	105.05	P18′	E25A	>1 mM
P3′	Q10A	13.55			
P14′	S21A	16.84			

**^2^** The numbering is according to the nomenclature of Schechter and Berger and using HNE as reference enzyme [18,19]. Important positions are indicated in Figure 1B.
